# Echocardiographic features, antithrombotic therapy, and association with outcomes in patients with left ventricular thrombus after myocardial infarction: a single-center retrospective study

**DOI:** 10.1007/s00392-026-03020-3

**Published:** 2026-09-30

**Authors:** Mariya Maslarska-Martens, Celina Blank, Tian Li, Marius Wessinger, David Hesselbarth, Diona Gjermeni, Kilian Franke, Christian Valina, Nikolaus Jander, Sebastian Grundmann, Dennis Wolf, Dirk Westermann, Christoph B. Olivier

**Affiliations:** 1 Department of Cardiology and Angiology, University Heart Center Freiburg – Bad Krozingen, Faculty of Medicine, University of Freiburg, Hugstetter Str. 55, 79106 Freiburg, Germany; 2 Stanford Center for Clinical Research, Department of Medicine, Stanford University School of Medicine Stanford, Stanford, CA USA

**Keywords:** Left ventricular thrombus, Acute myocardial infarction, Anticoagulation therapy, Vitamin k-antagonists, Direct oral anticoagulants

## Abstract

**Introduction and aim:**

Left ventricular thrombus (LVT) is a rare but threatening complication after acute myocardial infarction (MI). Guidelines recommend oral anticoagulation for at least 3–6 months.

This retrospective, single-center cohort study describes the incidence of LVT, the antithrombotic regimen, and the association with LVT regression and clinical outcomes.

**Methods:**

Patients admitted between January 1st, 2008, and December 31st, 2022, with MI were screened for the presence of LVT. Primary outcomes were the incidence of LVT, secular trends of oral anticoagulation prescription at discharge (direct oral anticoagulants vs Vitamin-K-antagonists), and change of LVT size or complete regression at follow-up examination. Secondary outcomes were all-cause death, embolic events associated with the LVT diagnosis, and bleeding events (BARC ≥ 2).

**Results:**

A total of 19,964 patients admitted with MI between 2008 and 2022 were identified, of whom 166 (0.83%) had a confirmed LVT. Eighty-five patients were prescribed a VKA, 49 patients a DOAC, and 26 patients another form of anticoagulation. Transthoracic echocardiography follow-up was available for 120 patients, of whom 95 (79%) showed complete thrombus resolution. No statistically significant difference in complete thrombus resolution was detected between DOAC and VKA groups (87% vs. 79%; *p* = 0.42). Thirty-eight clinical events (bleeding, all-cause death, and embolic events) occurred within 12 months after LVT diagnosis without significant differences between DOAC and VKA.

**Conclusion:**

DOAC use increased over time, with no statistically significant difference in early complete thrombus resolution or clinical events compared with VKA.

**Graphical abstract:**

**Figure:**
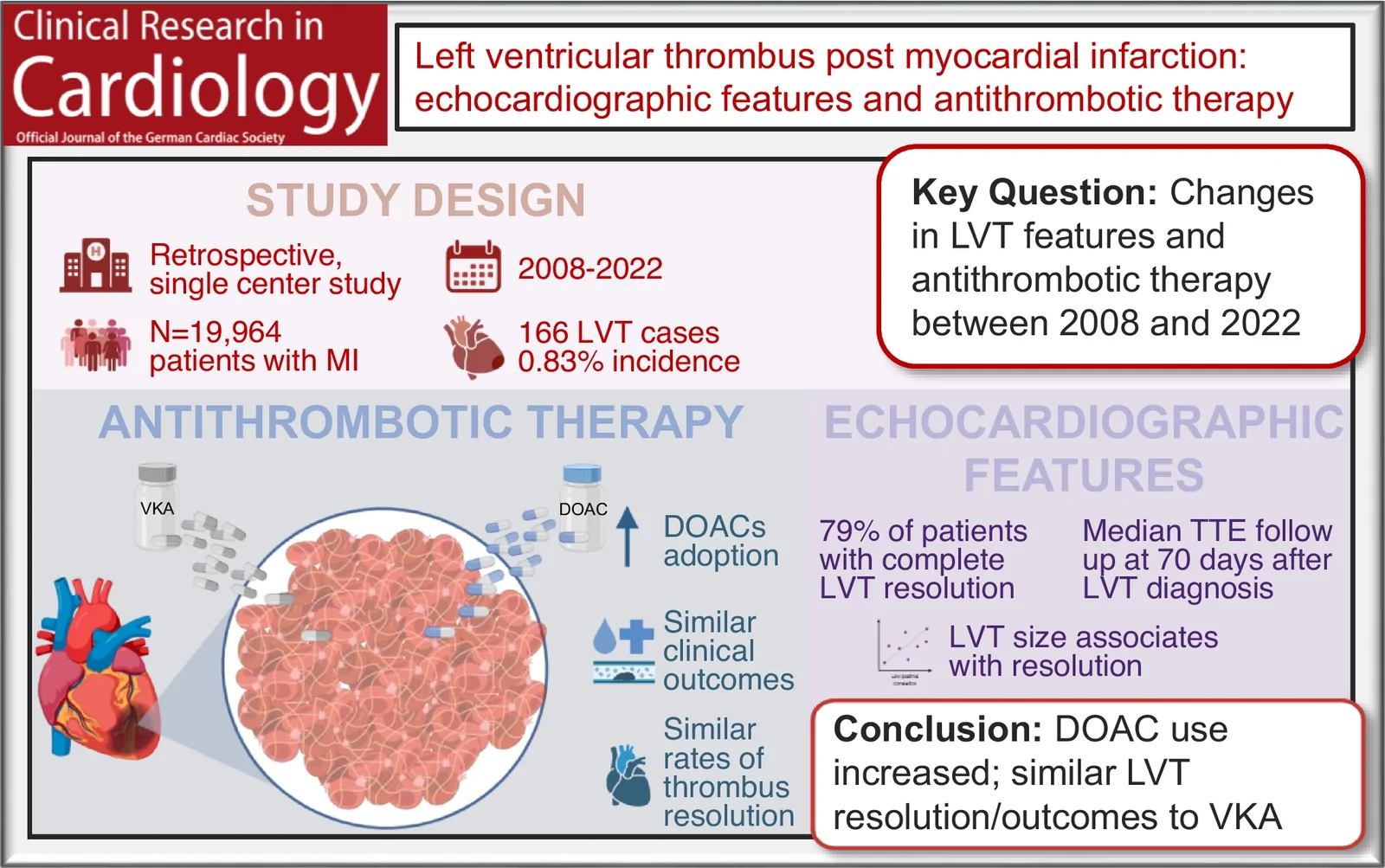

**Supplementary Information:**

The online version contains supplementary material available at https://doi.org/10.1007/s00392-026-03020-3.

## Introduction

Left ventricular thrombus (LVT) is a severe complication in patients with myocardial infarction (MI), especially in those with anterior ST-segment elevation myocardial infarction (STEMI) and impaired left ventricular systolic function [1, 2]. The reported incidence after MI ranges between 5 and 12% with a declining trend due to improved availability of coronary reperfusion therapy [3, 4]. Risk factors for the formation of LV-thrombi include additionally large myocardial injury, delayed coronary reperfusion, and presence of left ventricular aneurysm or anterior wall motion abnormalities [2, 3]. Early detection of LVT and initiation of anticoagulation are crucial to prevent systemic embolism [4]. The diagnostic pathway includes transthoracic echocardiography (TTE) often combined with contrast agent for better visualization of the apex and cardiac MRI with gadolinium contrast as gold standard with sensitivity up to 88% and specificity of 100% [4]. Current guidelines recommend therapeutic anticoagulation 3–6 months and repeat cardiac imaging to evaluate the current thrombus size or regression [5, 6]. The decision between a vitamin-K antagonist (VKA) and direct oral anticoagulants (DOACs) should be based on the individual bleeding risk and current antiplatelet therapy [6]. Data from a limited number of randomized controlled trials suggest the use of DOACs as non-inferior to VKA regarding thrombus regression and safety outcomes [7, 8]. There is a critical need to better define the incidence and evaluate the current therapeutic recommendations and clinical consequences of LVT in patients after acute myocardial infarction. This retrospective study aimed to describe the incidence of LVT in a tertiary cardiology hospital as well as the antithrombotic regimen and assess the LVT regression and clinical outcomes under the contemporary therapy.

## Methods

### Study design

This retrospective, single-center cohort study analyzed data from in-patients at the Department of Cardiology and Angiology, University Heart Center Freiburg-Bad Krozingen, Germany. The protocol was approved by the local ethics committee, privacy and security offices (No. 23–1338-S1-retro). Because of the retrospective character and anonymization of patient data no informed consent was required. The study was registered in the German Clinical Trials Register (DRKS00032548).

### Study population

All patients admitted to the Department of Cardiology of the University Heart Center Freiburg-Bad Krozingen between January 1 st 2008 and December 31 st 2022 were screened for ICD-10-code for MI (ICD-10-I21) to obtain the total number of patients admitted with this diagnosis. Patients were screened for ICD-10-code for intracardial thrombus without additional specification (I51.3) and intracardial thrombus in the atrium, auricular appendage or ventricle as a current complication after acute MI (I23.6) [9]. Diagnosis was confirmed by review of discharge letters and imaging documents. Furthermore, we performed a free text search in the patient management program using following keywords “lv thrombus”, “lv thrombi”, “left ventricular thrombus”, “left ventricle thrombus”, “apical thrombus” and “apical thrombi”. Clinical and diagnostic follow-up was based on data (discharge letters, written documentation of phone visits with patients after discharge and 12 months later, diagnostic findings) in the patients’ management program 12 months within LVT confirmation. The study period started in 2008 to coincide with the European Medicines Agency’s (EMA) authorization of dabigatran as first DOAC [10].

The main inclusion criteria were age of at least 18 years at the time of MI diagnosis, acute MI before the detection of LVT and confirmation of LVT by transthoracic echocardiography, cardiac MRI or CT-scan. Only patients with LVT diagnosed at the University Heart Center Freiburg-Bad Krozingen were included in the statistical analysis. Patients were excluded from the study if the MI or LVT diagnosis were only suspected without written confirmation or if the left ventricular thrombus was removed surgically.

### Outcomes, data analysis and statistical considerations

Primary outcomes were defined as the incidence of LVT after MI, rate of prescribed oral anticoagulation therapy (DOAC vs Vitamin-K-antagonists) and change of LVT size or complete LVT regression under the oral anticoagulation therapy with DOACs or VKA based on a follow-up examination with TTE. Secondary outcomes included all-cause death, embolic events associated with the LVT diagnosis and bleeding events defined by the Bleeding Classification System Definition [11]. Only bleeding events defined as BARC 2 or higher based on the hospital documentation were included in the statistical analysis. Clinical events were reviewed and assessed by a board-certified physician. Clinical outcomes were assessed individually and combined for the two anticoagulation strategies (DOAC vs Vitamin-K antagonists).

The electronic database included patients’ characteristics, medical history, initial and follow-up TTE and other radiological examination if performed (cardiac MRI/CT-scan), antithrombotic and anticoagulation therapy and clinical outcomes 12 months within LVT diagnosis. Missing echocardiographic measurements were completed by a board-certified physician trained in echocardiography.

The incidence of LVT for the total number of patients admitted with a MI at the University Heart Center Freiburg-Bad Krozingen in the period 2008–2022 was calculated. Patients were grouped according to the prescribed therapy at discharge. To investigate changes in prescribing patterns, the 15-year study period was divided into 3-year intervals, calculating the relative proportion of VKA and DOAC prescriptions for each period.

Variables are presented as median (interquartile range) and number (percent). The groups were tested for normal distribution using the Shapiro–Wilk adjustment test. Categorical variables were compared with Fisher’s exact test. Continuous variables were compared with a 2-sided unpaired *t*-test or Mann–Whitney *U*-test, depending on the normal distribution. Univariate and multivariate regression models to predict LVT resolution were created. The time to events (death, embolism, bleeding) within the first year after diagnosis of LVT was analyzed with Cox regression. The significance level was set at 0.05 (2-sided) for all analyses. IBM SPSS Statistics Version 29.0.0.0 (IBM SPSS Statistics, New York, USA) and GraphPad Prism Version 10.1.1 (GraphPad Software, Boston, Massachusetts USA) were used for the statistical analysis.

## Results

### Patient population

**Fig. 1:**
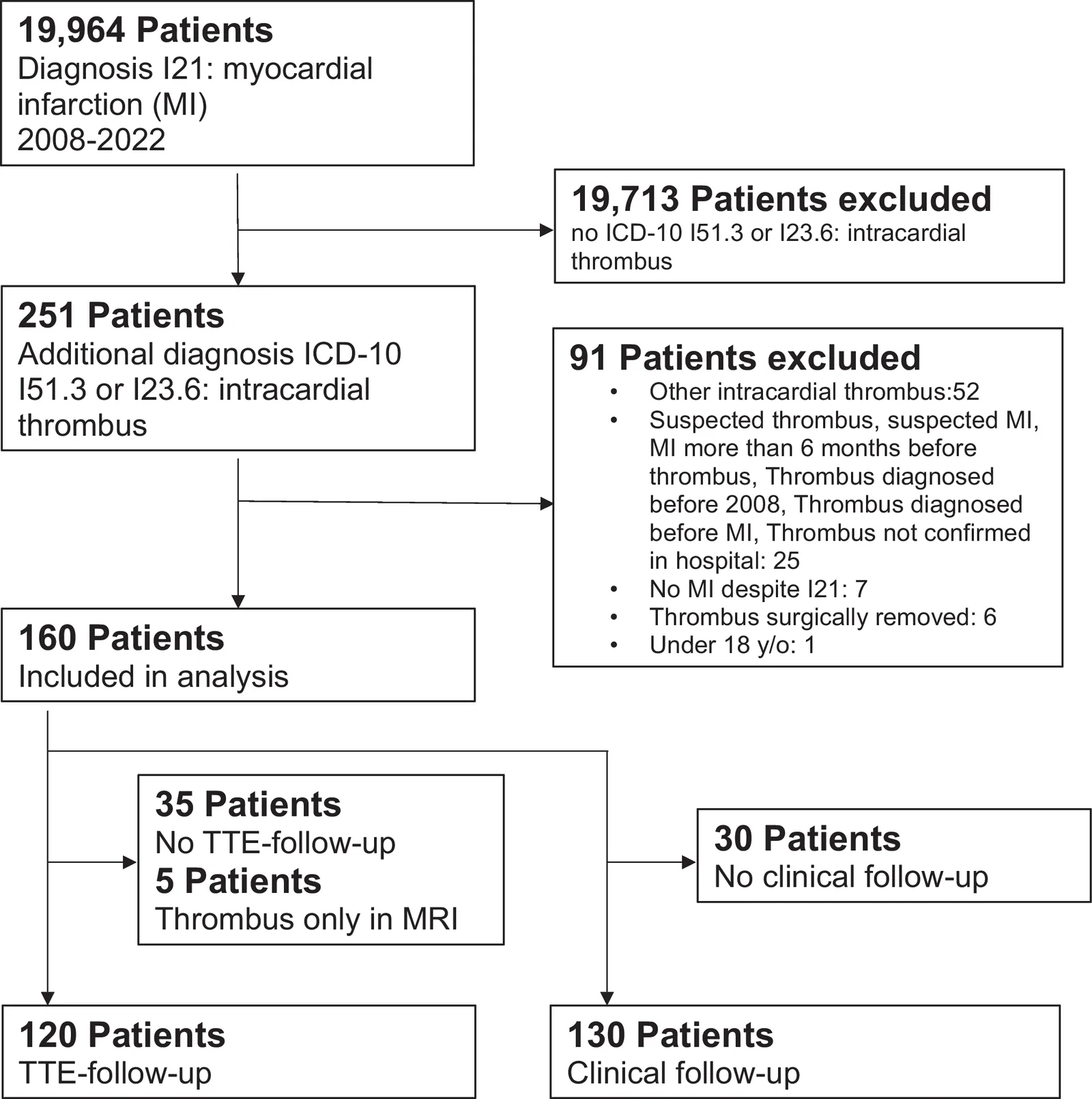
Flowchart for the detailed screening process and patients’ identification

19,964 patients admitted with an acute MI (ICD-10-I21) at the University Heart Center Freiburg-Bad Krozingen between January 1 st, 2008 and December 31 st, 2022 were identified. Two hundred fifty-one patients from this initial cohort presented an additional diagnosis for intracardial thrombus with ICD-10-I51.3 and/or ICD-10-I23.6. 25 patients were identified by the ICD-10-I23.6 code and 224 patients by the ICD-10-I51.3 code. Two patients were assigned both of the above mentioned ICD-10 codes. This subcohort was further screened for the presence of a left ventricular thrombus, resulting in 166 confirmed LVT cases. Six patients with LVT underwent surgical removal of the LVT and were excluded from the further statistical evaluation. The final study cohort included 160 patients. One hundred twenty patients were followed up with a transthoracic echocardiography within 12 months of LVT confirmation. A clinical follow-up based on hospital data was available for 130 patients, while 30 patients were lost to clinical follow-up. Figure 1 depicts the detailed screening process.

Of the 160 patients included for this analysis, 125 (78%) were male patients. The median age was 64 years (IQR 55–74). Twenty-five (16%) patients had a history of prior MI, ischemic stroke or transient ischemic attack (TIA) or gastrointestinal or intracranial bleeding. One hundred eighteen (74%) of all patients were admitted with STEMI. One hundred seven (67%) patients had an anterior localization. Six patients were referred for coronary artery bypass grafting (CABG). Eighteen patients received conservative therapy without coronary intervention or surgery. The median left ventricular ejection fraction (LVEF) in the initial TTE examination after MI was 35% (IQR 26–43). One hundred forty-one (88%) patients were diagnosed with one LVT. One hundred forty-three (89%) of all LVT cases diagnosed were located in the apex and the majority of LVT (67.5%) were characterized as organized. LVTs had a median diameter measured of 20 mm (IQR 14–26). A TTE contrast agent was applied in 11 patients (7%). The patients’ characteristics are summarized in Table 1. Forty-nine patients (31%) were discharged with a DOAC as anticoagulation therapy after LVT, whereas 85 patients (53%) received VKA. Twenty-six patients (16%) were prescribed other forms of anticoagulation such as heparin or other forms of antithrombotic therapy upon discharge. Sixty-one patients (48%) were discharged on OAC combined with single antiplatelet therapy (SAPT), whereas 66 (52%) received OAC with dual antiplatelet therapy (DAPT). Thirty-three patients (21%) did not receive any additional antiplatelet or anticoagulation therapy. The majority of patients received clopidogrel (91%) followed by prasugrel (5%) and ticagrelor (4%).

**Table 1:** Patients’ characteristics. Values are *n* (%), or median (IQR)

Characteristics	*N*=160	(%)/(IQR)
Male Sex	125	(78)
Age (years), median (IQR)	64	(55–74)
Prior coronary artery disease	20	(12.5)
Prior MI	14	(9)
Prior ischemic stroke or TIA	10	(6)
Prior gastrointestinal or intracranial bleeding	1	(1)
**Coronary angiography and PCI at time of MI diagnosis**		
STEMI	118	(74)
Anterior STEMI	107	(67)
PCI as initial therapy	136	(85)
LAD-PCI	119	(74)
**Initial TTE at LVT diagnosis**		
Left ventricle ejection fraction (LVEF) (%), median (IQR)	35	(26–43)
LVT diameter in TTE (mm), median (IQR)	20	(14–26)
LVT characterized as mural	108	(67.5)
LVT localization in the apex	143	(89)
Use of TTE contrast agent of LVT detection	11	(7)
**Antithrombotic therapy at discharge**		
Dual antithrombotic therapy (DAT)	61	(38)
Triple antithrombotic therapy (TAT)	66	(41)
No antiplatelet or anticoagulation therapy upon discharge	33	(21)
**Type of anticoagulation at discharge**		
VKA	85	(53)
Other forms of antithrombotic therapy (e.g. heparin)	26	(16)
DOAC	49	(31)
**Type of DOAC**		
Apixaban	9	(6)
Rivaroxaban	33	(21)
Dabigatran	5	(3)
Edoxaban	2	(1)

*IQR* interquartile range; *LVT* left ventricular thrombus; *MI* acute myocardial infarction; *TIA* transient ischemic attack; *STEMI* ST-segment elevation myocardial infarction; *LAD* left anterior descending artery; *PCI* percutaneous coronary intervention; *TTE* transthoracic echocardiography; *OAC* oral anticoagulation therapy; *VKA* vitamin-K antagonists; *DOAC* direct oral anticoagulants

### Primary outcomes

**Fig. 2:**
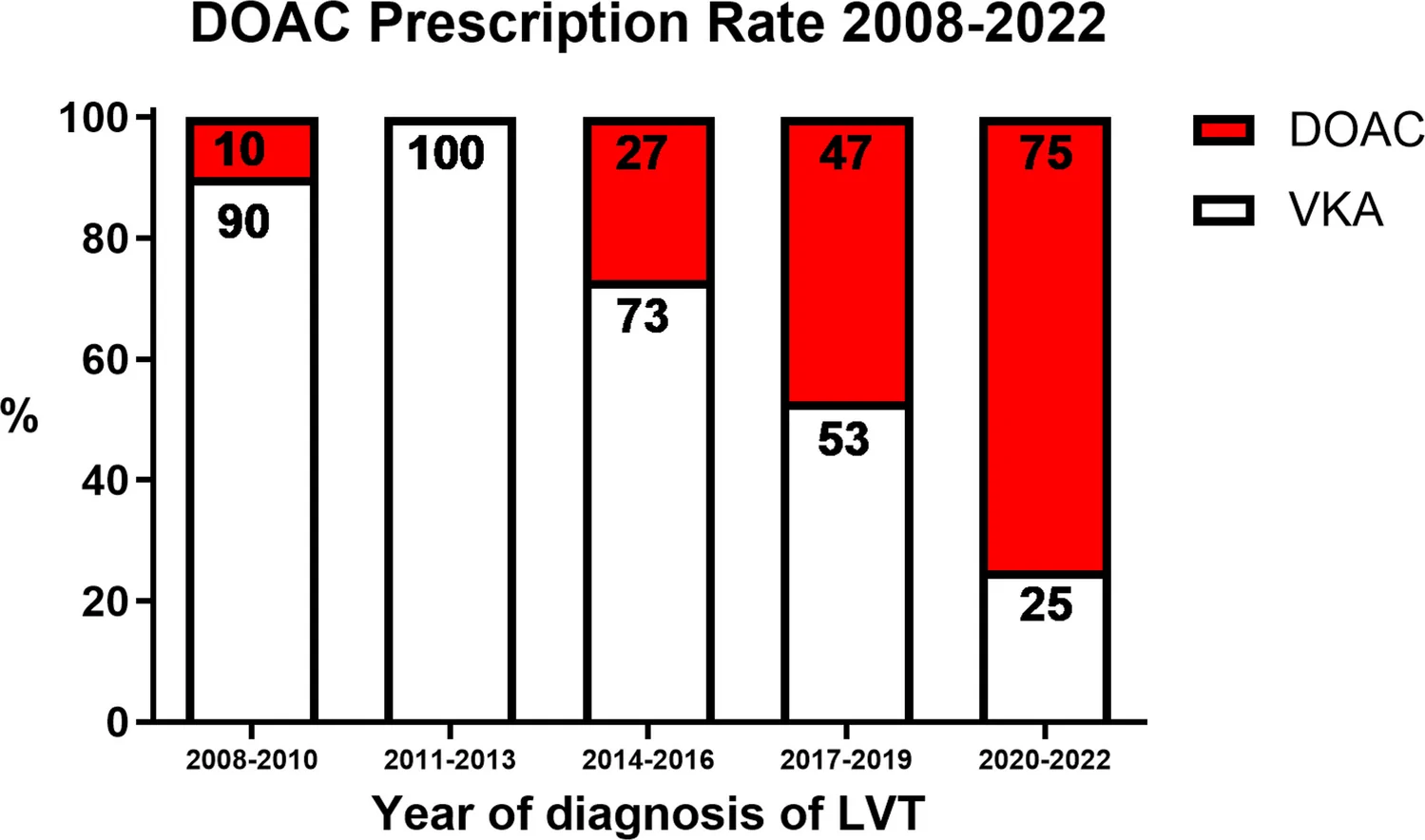
Changes in the prescribed anticoagulation therapy in a tertiary cardiology hospital between 2008 and 2022 with a rise in DOAC prescription over time (*p* < 0.001)

The observed hospital-based detection rate of LVT after acute MI was 0.83% (166/19,964) between January 1 st 2008 and December 31 st 2022. The prescription rates between DOAC and VKA shifted throughout the years with a prescription rate between 73 and 100% for VKA. between 2008 and 2016. After 2016, DOAC use gradually increased (prescription rate of 47% between 2017 and 2019 and 75% between 2020 and 2022 (*p* < 0.001, Fig. 2).

The median duration between the first TTE to confirm the LVT diagnosis and follow-up examination was 70 days (IQR 19–154) for all patients. Patients with complete LVT resolution were followed up with a TTE after a median of 89 days (IQR 34–166) after LVT confirmation. Patients who were diagnosed with a persistent LVT in the follow-up TTE were examined after a median of 25 days (IQR 5–113) after the initial diagnosis. Of all 120 patients with a TTE follow-up, 25 patients (21%) were diagnosed with a persistent LVT, and 95 patients (79%) demonstrated complete LVT resolution. In 20 out of these 25 persistent LVT, thrombus size was measured during the first and the follow-up examination. The majority of LVT (70%) demonstrated a decrease in size with a median reduction of –5.75 mm (IQR − 11.85–1).

**Fig. 3:**
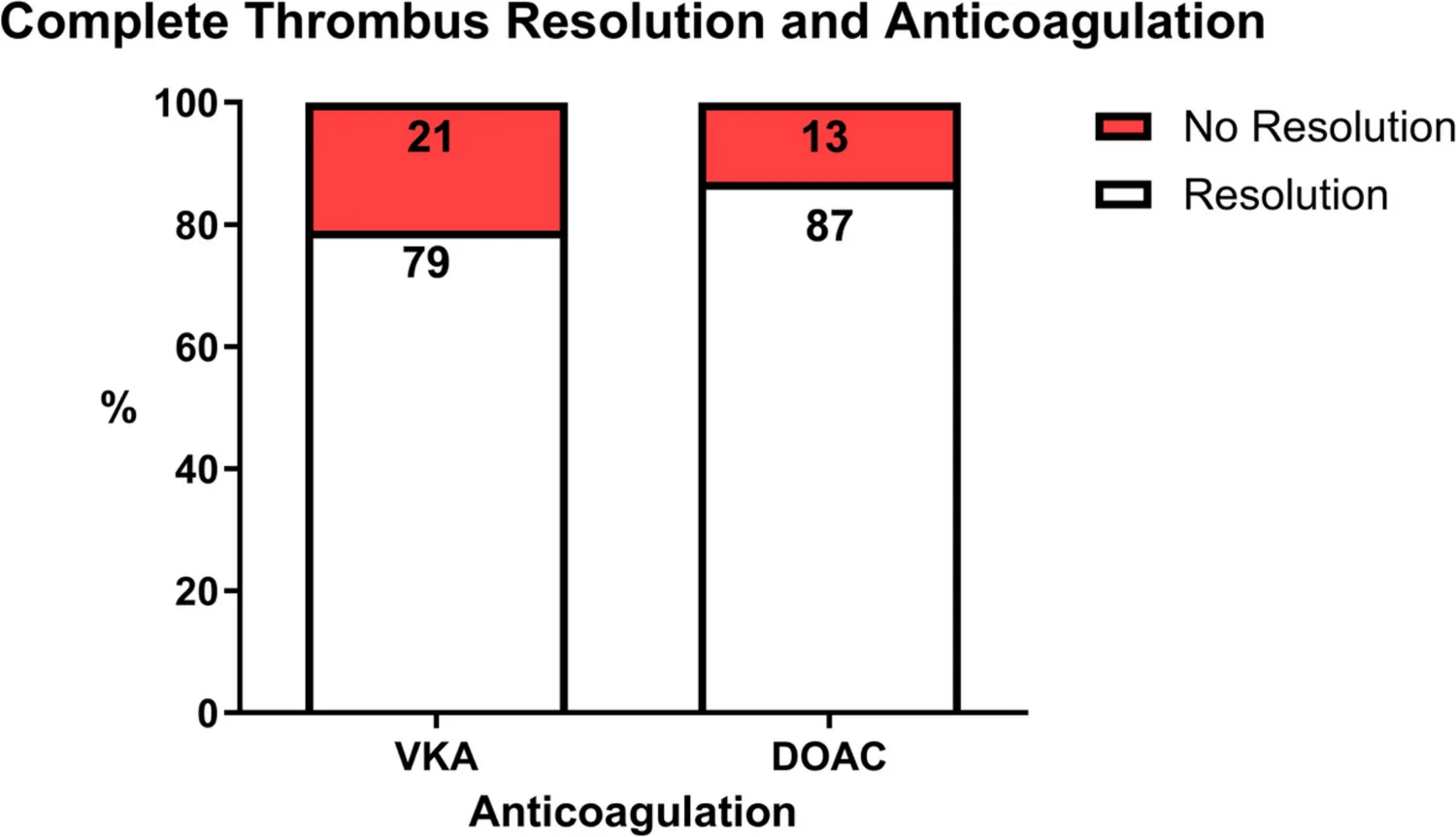
Left ventricular thrombus resolution under oral anticoagulation therapy with no significant difference between DOAC and VKA (*p* = 0.42)

In the subgroup analysis regarding the type of anticoagulation, 79% of LVT presented complete resolution under therapy with VKA. In the DOAC subgroup 87% of LVT fully resolved under the anticoagulation (*p* = 0.42, Fig. 3). Patients discharged with DOAC therapy were followed-up after a median of 103 days (IQR 49–187). Patients receiving VKA upon discharge were re-examined after a median of 65 days (IQR 29–158). A subgroup analysis between dual antithrombotic therapy (DAT) and triple antithrombotic therapy (TAT) depicted similar rates of LVT resolution (DAT: 81.8% resolution, TAT: 82.3% resolution, *p* = 0.946).

**Fig. 4:**
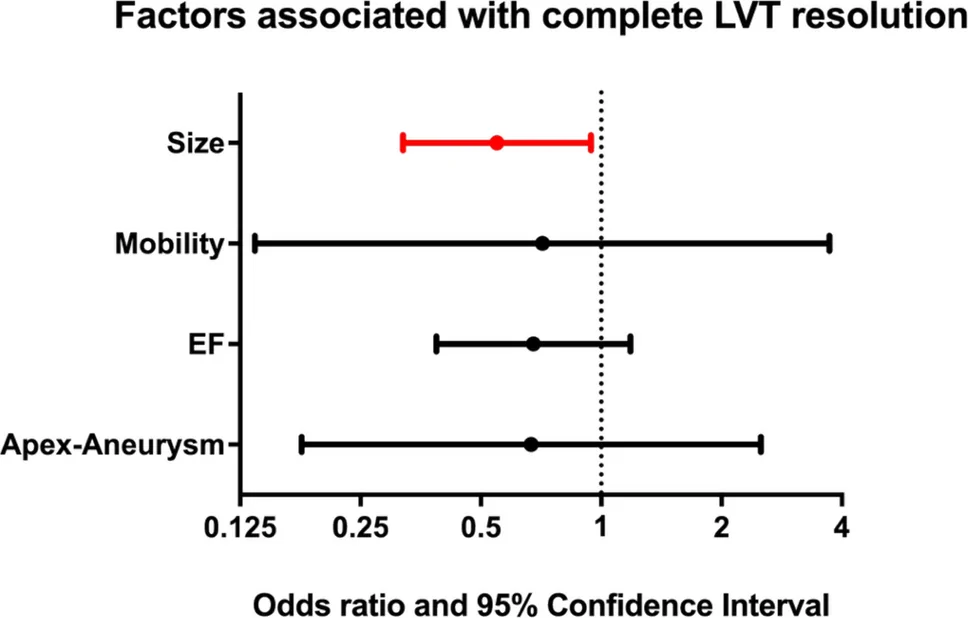
Factors associated with complete thrombus resolution. Initial thrombus size was the only significant predictor for LVT resolution. The multivariate regression model with all possible factors for complete LVT resolution was not significant (*p* = 0.087). EF, ejection fraction

A logistic regression model for factors associated with LVT resolution with thrombus size at the time of diagnosis, thrombus characterization, LVEF and presence of apex aneurysm at the time of LVT diagnosis was created. Initial thrombus size was predictive for thrombus resolution at follow-up with an odds ratio (OR) of 0.55 (95% CI 0.32–0.94) (*p* = 0.030). Thrombus characterization (*p* = 0.688), LVEF (*p* = 0.170), and presence of apex aneurysm (*p* = 0.550) were not predictive for LVT resolution at follow-up in the multivariable model. The regression model was statistically not significant (*p* = 0.087). Figure 4 depicts the multivariable regression model with each variable as a predictor.

### Secondary outcomes

**Fig. 5:**
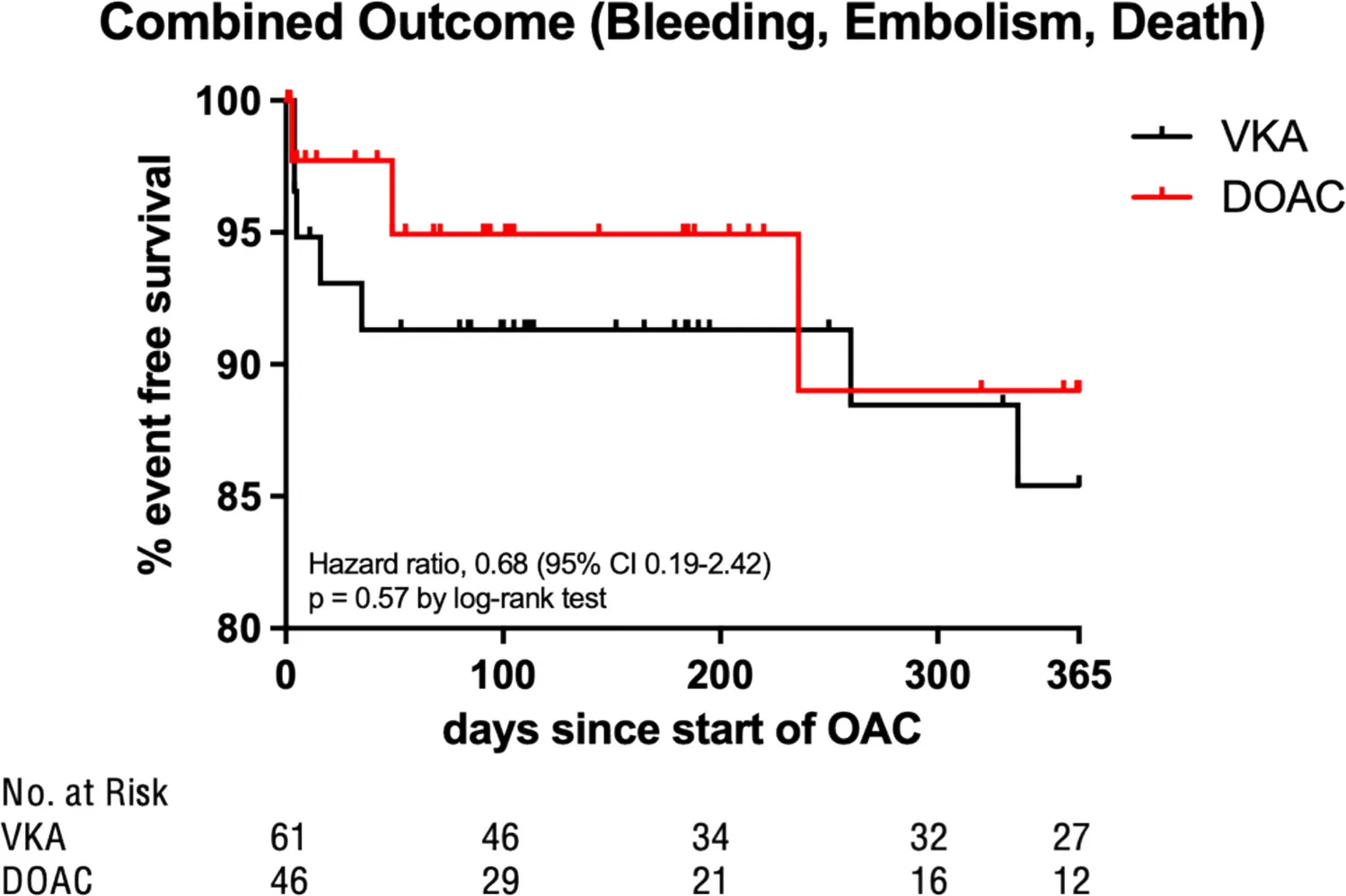
Kaplan–Meier curve the combined outcomes (bleeding, embolism, death) between DOAC and VKA. No significant difference between survival rates in the first year after OAC start according to the OAC strategy [HR 0.68 (95% CI 0.19–2.42); *p* = 0.57]

Based on the clinical follow-up during the 12 months after LVT diagnosis, we identified 14 embolic events, 8 bleeding events (BARC ≥ 2) and 16 deaths in a total of 25 patients. Embolic events were documented shortly after the LVT diagnosis after a median of 2.5 days (IQR 1–6.5). Bleeding events were observed after a median of 51 days (IQR 41–348). Death was reported after a median of 6 days (IQR 4–93.5) after diagnosis confirmation. In the subgroup analysis for DOAC and VKA, patients in the VKA group experienced 7 clinical events (3 death cases, 2 embolic events and 2 bleeding events) compared with 3 clinical outcomes (1 embolic and 2 bleeding events) in patients, receiving DOACs. No statistically significant difference in the survival rates between patients with VKA or DOACs after LVT diagnosis was observed (HR 0.68 (95% CI 0.19–2.42), *p* = 0.57; Fig. 5). Clinical outcomes in patients discharged with DAT did not differ from those in patients with TAT with no deaths and 1 embolism in patients on DAT, and 3 deaths and 2 embolisms in patients on TAT (HR 0.34 (95% CI 0.1–1.2), *p* = 0.09). Two bleeding events were documented in each subgroup during the observation period.

## Discussion

In this retrospective, single-center cohort study 0.83% of patients admitted with MI had a documented LVT. Predominant anticoagulation prescription switched from VKA to DOAC during the last 15 years. The thrombus resolution rate in this study was with 79% high irrespective of the anticoagulation therapy and thrombus size at diagnosis predicted resolution. Embolic events, all-cause death and bleeding were not statistically different between patients prescribed VKA compared with those prescribed DOACs.

This study cohort consisted of almost 20,000 initially screened patients from a tertiary center. The number of screened patients is substantially higher compared to other cohorts in retrospective and prospective studies from 2012 to 2022 [3]. Patients with confirmed LVT included in the statistical analysis did not differ from patient groups in previous studies with the majority being male and presenting with anterior STEMI [3]. The screening process might have resulted in a certain degree of selection bias due to the retrospective character and LVT diagnosis based on ICD-10 codes. However, this study cohort is still representative due to its size and screening duration of 15 years.

This study demonstrated lower incidence of LVT after acute myocardial infarction of 0.83% for a tertiary cardiology hospital with 19,964 patients admitted with MI between 2008 and 2022. This incidence is lower than the previously described LVT rate of 5–12% after MI [3, 4]. Patients examined with TTE without contrast agent demonstrate an LVT incidence of 4.3% in prior publications. Patients in this study were immediately transferred for coronary angiography upon admission with a median duration between admission and coronary angiography of 0 days. Patients received their first transthoracic echocardiography after a median of 2 days (IQR 0–5) after admission. The hospitalization period for STEMI and NSTEMI did not differ significantly with a median duration of 8 days. After discharge patients were referred to an outpatient cardiologist with a follow-up examination in the hospital based on the individual medical history. Literature suggests that formation of LVT takes place in the first 2 weeks after MI and the diagnostic sensitivity increases over time [6, 12]. In this way, a short-term follow-up examination could play an essential role for the detection and confirmation of the diagnosis. Furthermore, the lack of delay between admission and coronary angiography played an important role for the low LVT incidence reported in this study.

This study demonstrated a significant shift in the prescription rate between DOACs and VKA over the course of 15 years with rising acceptance for DOACs. The latest ESC Guidelines for the management of acute coronary syndromes recommend the use of DOAC or VKA based on the individual patient’s history with no specific preference for the medication [6].

Findings regarding thrombus resolution and clinical outcomes under anticoagulation therapy are consistent with results from contemporary randomized controlled trials (RCTs), which have recruited a limited number of patients. In their latest RCT with 50 participants, Jenab et al. demonstrated a better rate of LVT resolution under rivaroxaban (76%) after 3 months compared to VKA (54%). There were no differences in the occurrence of major bleeding or all-cause death between the anticoagulation subgroups [8]. Similar trials with a small number of participants between 35 and 79 patients [7, 13] reported about the non-inferiority of DOACs as opposed to VKA. In the beginning of 2025, an RCT with 261 patients showed similar rates of LVT resolution (95.8% for rivaroxaban and 96.6% for VKA) at the 3-month follow-up. All-cause mortality, rates of ischemic stroke and major bleeding were comparable in the subgroups [14]. Our findings support the available randomized data by demonstrating comparable rates of thrombus resolution and adverse events between DOAC and VKA. The subgroup analysis in patients discharged with DAT or TAT did not show differences in thrombus resolution and clinical events. Patients who require antiplatelet therapy after MI and have an indication for anticoagulation therapy for atrial fibrillation for example, exhibit a higher risk for major bleeding events [15, 16]. The use of DOACs in these patients is associated with a lower bleeding risk and similar efficacy compared to VKA [17]. The findings reported in the existing literature could be extrapolated for patients with LVT after MI with indication for both OAC and antiplatelet therapy. The results from the subgroup analysis, regarding SAPT and DAPT, are in line with the previously mentioned results from randomized trials that rates of thrombus resolution and serious clinical events for DOACs are similar to VKA in patients with LVT when additional antiplatelet therapy is necessary.

This retrospective study explored the echocardiographic characteristics of LVT in the beginning and during follow-up by creating a prediction model for LVT resolution. Even though the cumulative regression model was not significant (*p* = 0.087), we found that initial thrombus size plays an essential role as a risk factor for LVT persistence under anticoagulation therapy. In this way, echocardiography should aim not only for accurate detection of LVT but furthermore for precise visualization and measurement. The use of contrast-enhanced TTE could assist this process as recommended in the guidelines [6]. Unfortunately, TTE contrast agent was applied in the minority of LVT cases, which could be explained with the screening period taking place before the latest guidelines recommendations.

### Strengths and limitations

This study represents a comprehensive and thorough overview of the LVT incidence and shift in anticoagulation strategies over 15 years. One of the main strengths is the large patient cohort of 19,964 patients reviewed. Imaging data such as TTE images and reports were available in a digital form so that bias through missing hospital data can be excluded. In addition, data was reviewed by two different staff members of the trial and missing echocardiographic data was evaluated by a board-certified physician, trained in echocardiography.

One of the main study limitations is the retrospective character. The ICD-10-I23.6 code for intracardial thrombus as a current complication of acute MI was applied in the minority of cases (11%). As a result, the reported incidence could be underestimated due to clinical coding inconsistencies. Study data, specifically the clinical outcomes, is based on the patients’ management program and documentation. Due to the study type, patients with confirmed LVT were not contacted by the study staff regarding anticoagulation therapy and clinical events. The results are based on the echocardiographic examinations in our center so we cannot exclude additional follow-up examinations in outpatient cardiology centers. The variation in follow-up periods for patients with persistent LVT and complete LVT resolution as well as those receiving DOAC or VKA upon discharge, could have introduced potential bias in the reported LVT resolution rate.

## Conclusion

This retrospective, single-center cohort study identified a low incidence of left ventricular thrombus after acute myocardial infarction in a high-volume cardiology center with almost 20,000 patients admitted with MI over the last fifteen years. DOAC prescribing increased ove time, without a significant difference in LVT resolution or occurrence of clinical events. LVT remains a serious complication after MI despite the declining incidence and improved coronary reperfusion therapy. In this way, precise initial diagnostic as well as short-term follow-up examinations contribute for the early detection and initiation of anticoagulation. This study is limited by its retrospective character with patient data based on hospital documentation and by the lack of direct clinical follow up visit with the patients.

These results support the increasing use of DOACs as anticoagulation therapy for LVT as well as the need for standardized follow-up protocols for these patients. Larger randomized controlled trials are necessary to confirm the safety and efficacy of DOACs. 

## Supplementary Information

Below is the link to the electronic supplementary material.

ESM 1 (DOCX 13.7 KB)
