# Supplementary material for: Echocardiographic features, antithrombotic therapy, and association with outcomes in patients with left ventricular thrombus after myocardial infarction: a single-center retrospective study
Source: Clin Res Cardiol. 2026 Sep 30;115(12):2241–50. doi: 10.1007/s00392-026-03020-3 (PMC13751566; doi:10.1007/s00392-026-03020-3)
Supplement: Supplementary file 1 [file 392_2026_3020_MOESM1_ESM.docx]

# SUPPLEMENT MATERIAL

**Definitions**

- First transthoracic echocardiography was defined as the first TTE examination after MI with LVT confirmation or TTE after MI at the same time of LVT diagnosis confirmed by CT-scan or cardiac-MRI
- Follow-up TTE
- First follow-up TTE with complete LVT resolution within 12 months of LVT confirmation
- In case of LVT persistence: last TTE with evidence of LVT within 12 months of LVT confirmation
- Antithrombotic therapy for the statistical analysis was defined as medication upon discharge after MI
- Start of oral anticoagulation therapy was defined as the first day of medication intake irrespective of bridging therapy (if prescribed ahead of oral anticoagulation)

**Patient screening**

- Patients were only identified through the ICD-10 codes I21 for myocardial infarction and I51.3 or I23.6 for intracardial thrombus
- No additional patients were identified through the free text search in the patient management program with following keywords: “ lv thrombus”, “lv thrombi”, “ left ventricular thrombus”, “ left ventricle thrombus”, “apical thrombus” and “apical thrombi”.
